# Landscape Diversity and Crop Vigor Influence Biological Control of the Western Grape Leafhopper (*E*. *elegantula* Osborn) in Vineyards

**DOI:** 10.1371/journal.pone.0141752

**Published:** 2015-11-10

**Authors:** Houston Wilson, Albie F. Miles, Kent M. Daane, Miguel A. Altieri

**Affiliations:** Department of Environmental Science, Policy and Management, University of California, Berkeley, California, United States of America; Fresno, UNITED STATES

## Abstract

This study evaluated how the proportional area of natural habitat surrounding a vineyard (i.e. landscape diversity) worked in conjunction with crop vigor, cultivar and rootstock selection to influence biological control of the western grape leafhopper (*Erythroneura elegantula* Osborn). The key natural enemies of *E*. *elegantula* are *Anagrus erythroneurae* S. Trjapitzin & Chiappini and *A*. *daanei* Triapitsyn, both of which are likely impacted by changes in landscape diversity due to their reliance on non-crop habitat to successfully overwinter. Additionally, *E*. *elegantula* is sensitive to changes in host plant quality which may influence densities on specific cultivars, rootstocks and/or vines with increased vigor. From 2010–2013, data were collected on natural enemy and leafhopper densities, pest parasitism rates and vine vigor from multiple vineyards that represented a continuum of landscape diversity. Early in the season, vineyards in more diverse landscapes had higher *Anagrus* spp. densities and lower *E*. *elegantula* densities, which led to increased parasitism of *E*. *elegantula*. Although late season densities of *E*. *elegantula* tended to be lower in vineyards with higher early season parasitism rates and lower total petiole nitrogen content, they were also affected by rootstock and cultivar. While diverse landscapes can support higher natural enemy populations, which can lead to increased biological control, leafhopper densities also appear to be mediated by cultivar, rootstock and vine vigor.

## Introduction

Natural habitats are an important source of ecosystem services to agriculture, including biological control of crop pests [1–3]. By providing important resources for natural enemies, such as refugia, overwintering habitat, nectar, pollen and alternate hosts or prey, non-crop habitats can support natural enemy populations in nearby crop fields, which can lead to increased levels of biological control of pests [4]. As such, changes in the area, density and species composition of natural habitats surrounding an agroecosystem can impact natural enemy diversity and abundance and their subsequent impact on crop pests, but outcomes are typically asymmetric due to differences in natural enemy dispersal ability and the degree to which a given species relies on non-crop resources [5–7].

Herbivore performance can also be strongly influenced by structural and chemical characteristics of the host-plant itself, which in agriculture is largely determined by crop cultivar and vigor, and in the case of woody perennial crops rootstock selection as well [8–10]. Of course, host-plant quality can also be influenced by environmental conditions, such as ambient temperature and water status. Trichomes and leaf hairs are a structural defense that interfere with insect movement [11] while the production of certain secondary metabolites in plant tissue that are toxic to herbivores act as a chemical defense [12, 13]. These traits are not exclusive, for instance glandular trichomes combine both structural and chemical defenses [14]. The presence or absence of such traits associated with a specific crop cultivar or rootstock can be a strong determinant of pest densities regardless of natural enemy impact [15].

Furthermore, changes in crop vigor can alter secondary plant metabolites in a way that can be beneficial to insect herbivores. The plant stress hypothesis suggests that reduced metabolism in drought stressed plants can lead to increased concentrations of soluble nitrogen in plant tissue and/or decreased production of chemical defense compounds, both to the benefit of herbivores [16, 17]. Alternately, the plant vigor hypothesis states that overly vigorous plants are also more preferable to insects due to the increased quality of newly developed tissue [18]. Crop vigor can be influenced by a number of factors, including soil fertility and moisture [19] as well as crop cultivar and rootstock selection [20–23]. Rootstocks in particular play an important role, as they mediate uptake of nutrients and water, as well as synthesis of key plant hormones [24]. Previous studies have demonstrated that herbivore preference and performance can be influenced by rootstock selection, this of course includes root feeding insects [25–27] but the effect extends to foliar feeders as well [28–30].

The western grape leafhopper (*Erythroneura elegantula* Osborn [Hemiptera: Cicadellidae]) is a key pest of wine grapes in California’s North Coast region. The adults overwinter in reproductive diapause, commonly inhabiting leaf litter or perennial vegetation found in and around the vineyard. Adults become active in the spring and, after feeding on fully expanded mature grape leaves, they break their reproductive diapause, mate and then the females begin to oviposit into grape leaves. In this wine grape region, *E*. *elegantula* typically completes two generations per year before grape vines senesce and the adults enter diapause and move back onto the vineyard floor [31].

The key parasitoids of *E*. *elegantula* are *Anagrus erythroneurae* S. Trjapitzin & Chiappini and *A*. *daanei* Triapitsyn (Hymenoptera: Mymaridae). Both species attack the eggs of *E*. *elegantula* as well as those of closely related leafhoppers. Spiders are the most abundant generalist predator in vineyards [32]. A number of additional generalist predators are also known to feed on *E*. *elegantula* eggs, nymphs and/or adults, including soldier beetles (Cantharidae), minute pirate-bugs (*Orius* sp.), green lacewings (*Chrysoperla* sp.), convergent lady beetle (*Hippodamia convergens* [Guérin-Méneville]), big-eyed bugs (*Geocoris* sp.), brown lacewings (*Hemerobius* sp.), damsel bugs (*Nabis* sp.), and hover fly larva (Syrphidae) [31]. Although generalist predators, and spiders in particular, can have an impact on *E*. *elegantula* densities [33, 34], *Anagrus* wasps are considered the dominant biological control agent in this system.

Key to the effectiveness of *Anagrus* as a parasitoid of *E*. *elegantula* is their different overwintering habitat requirements. While adults of *E*. *elegantula* can successfully overwinter in and around vineyards, *Anagrus* must seek out and parasitize an alternate leafhopper host species that overwinter in the egg stage. These alternate hosts are typically found in natural and semi-natural habitats located outside of vineyards [35–37]. As such, there is seasonal movement of *Anagrus* between cultivated wine grapes (where they attack *Erythoneura* spp. from April—September) and natural habitats (where they attack alternate leafhopper hosts to overwinter from October—March). Insufficient overwintering habitat near the vineyard for these alternate leafhopper hosts could result in the later arrival and/or lower abundance of *Anagrus* wasps colonizing the system each year, which could in turn have negative implications for biological control of *E*. *elegantula*.

While a number of studies have evaluated the influence of adjacent patches of natural and semi-natural habitat (e.g., French prunes) on natural enemies and biological control of vineyard leafhoppers [38–42] only a few have evaluated the influence of landscape diversity on natural enemies [43–46], and none have measured natural enemy impact on leafhoppers. The aim of this study was to evaluate whether or not natural enemy populations and biological control of *E*. *elegantula* are influenced by changes in the proportion of natural habitat surrounding the vineyard; however, the analyses also considered the effects of grape cultivar, rootstock and vine vigor on *E*. *elegantula* densities. This was achieved by collecting data over a four year period (2010–2013) on *E*. *elegantula* and natural enemy densities, parasitism rates and vine vigor from 33 vineyards that represented a continuum of landscape diversity in one geographic area. Data generated from this study will provide insight into the key factors driving the relationship between landscape diversity and biological control of *E*. *elegantula* in vineyards.

## Materials and Methods

### Study Sites

Field sites consisted of vineyard blocks >0.4 hectares (1 acre) located in Napa and Sonoma County, California, USA. There were 21, 25, 17, and 7 sites in 2010, 2011, 2012, and 2013, respectively. The vineyards were situated in low, intermediate and high diversity landscapes, as measured by the relative proportion of non-crop natural habitat within 0.5 km. Permission to access all vineyard sites was given by the land owners.

All vineyard blocks were located on flat, level ground and consisted of vines that were red cultivars at least five years old. Cultivars were mostly Cabernet Sauvignon but also included Merlot, Pinot Noir, Zinfandel and Petit Verdot. Rootstocks included 101–14, 5C, O39-16, 3309C, St. George, 44-53M, 110-R, 1103P and Schwarzmann. Specific cultivar-rootstock combinations at the study sites can be found in Table 1. The vigor of these cultivars and rootstocks varies and the cultivars also exhibit differences in leaf hair density, two factors that can influence leafhopper host-plant preference. See Table 2 for details about these key characteristics for the cultivars and rootstocks in this study. Each experimental block was comprised of 40–80 vine rows with 50–80 vines per row. All samples were taken from 5 vine rows in the middle of the block. Within each of the sample rows no measurements were taken from the first or last ten vines.

**Table 1:** Key Characteristics of Cultivars and Rootstocks at the Study Sites [47].

Cultivar / Rootstock	*Vitis* Parentage	Vigor	Leaf Hairs
Cabernet Sauvignon	*vinifera*	High	Sparse
Merlot	*vinifera*	Medium—High	Sparse
Pinot Noir	*vinifera*	Low—Medium	Dense
Petit Verdot	*vinifera*	Medium—High	Dense
Zinfandel	*vinifera*	Medium	Dense
101–14 Mgt	*riparia* x *rupestris*	Medium	-
110R	*berlandieri* x *rupestris*	Medium	-
1103P	*berlandieri* x *rupestris*	Medium—High	-
3309C	*riparia* x *rupestris*	Medium	-
44-53M	*riparia* x (*cordifolia* x *rupestris*)	Medium	-
5C	*berlandieri* x *riparia*	Low—Medium	-
O39-16	*vinifera* x *rotundifolia*	High	-
Schwarzmann	*riparia* x *rupestris*	Medium	-
St. George	*rupestris*	High	-

**Table 2:** Number of Study Sites with Specific Cultivar-Rootstock Combinations.

	Merlot	Cabernet Sauvignon	Pinot Noir	Petit Verdot	Zinfandel
**5C**	5	4			
**O39-16**		2			
**3309C**	2	1	1		
**St. George**		2			2
**101–14 Mgt**	1	6	1	1	
**44-53M**		1			
**Schwarzmann**		1			
**110R**		2			1
**1103P**		1			

Throughout the course of the study no insecticides targeting leafhoppers or other pierce-suck pests were used at any of the study sites. Insecticides were applied at a subset of sites as part of a mandatory eradication program for the invasive European grapevine berry moth (Tortricidae: *Lobesia botrana* Denis & Schiffenmüller) in 2010–2012 [48]. These sprays took place at 10 of the 21 sites in 2010, 14 of the 25 sites in 2011, 17 of the 17 sites in 2012, and 0 of the 7 sites in 2013. All sprays consisted of non-contact products, including insect growth regulators, diamides, microbial insecticides (e.g. *Bacillus thurigiensis*), avermectins and spinosyns. With the exception of spinosyns, these pesticides have low natural enemy toxicity [49]. While spinosyns can be moderately toxic to parasitoids [50], their use was restricted to just one spray at one of the study sites in 2010 and 2011. All of these products have little to no impact on *E*. *elegantula* populations [49].

### Quantification of Landscape Diversity

Landscape diversity was quantified by extracting “rangeland cover type” from the CalVEG dataset (USDA Forest Service Remote Sensing Lab) using ArcGIS 10.1 (ESRI, Redlands, USA). There were 71 possible values for rangeland cover type, as described by Shiflet [51]. The total area of each cover type was calculated within a 500 m radius around each vineyard site. Cover types were then consolidated into five categories: “natural habitat”, “agriculture”, “development”, “water” and “no data”. The “natural habitat” category consisted primarily of riparian, oak woodland and chaparral habitats while the “agriculture” category was almost entirely vineyard. “Development” included all commercial and residential areas, including urban vegetative landscaping. For this analysis, “landscape diversity” is defined as the percentage of “natural habitat” within 500 m of the vineyard study site.

### Insect Abundance and Parasitism Rate

#### Natural enemy and *E*. *elegantula* adult abundance

Yellow sticky-traps were used to monitor the abundance of *Anagrus* wasps, key generalist predators and *E*. *elegantula* adults in the early season (April 29 –June 4 2010; April 13 –May 25 2011; April 24 –June 6 2012; April 12 –May 22 2013) and late season (July 6 –August 18 2010; July 14 –September 7 2011; July 10 –August 21 2012; July 14 –August 28 2013). These early and late season periods approximately coincided with the peak adult flight period for first and second generation *E*. *elegantula* populations. At each vineyard, five yellow sticky-traps (16 x 10 cm; Seabright Laboratories, Emeryville, CA) were randomly assigned to vines within the sampling area and hung in the vine canopy from a trellis wire. Traps were replaced approximately every two weeks.

#### Leafhopper egg parasitism rate

Leafhopper egg parasitism was determined by evaluating 30 grape leaves from each site. Leaves were collected by removing 1 leaf each from 30 randomly selected vines. Parasitism rates were assessed twice each season following peak nymph density of the first generation (~1–15 June) and second generation (~20 July–15 August) at each site. Leaves were collected from shoot nodes one to three for the first generation and nodes four to six for the second generation. Leaves were brought to the laboratory and inspected with a dissecting microscope. Egg status was determined by the emergence mark present–a small slit in the egg close to the leaf surface indicates that *E*. *elegantula* had successfully emerged while a circular hole on the top of the egg indicated emergence of an *Anagrus* wasp. Unemerged eggs were not included in the parasitism assessment, as their status could not be consistently determined.

#### Spiders in the vine canopy

Following Costello and Daane [32], spiders were sampled from the vine canopy using a modified beat-sheet in August or September of each year (2 September 2010, 11 August 2011, 12 September 2012 and 15 September 2013). The beat-sheet consisted of a 1 m^2^ cloth funnel that fed into a detachable 3.78 liter (1 gallon) plastic bag. Samples were collected from five randomly selected vines at each vineyard site. Sampling involved holding the funnel beneath the vine canopy and vigorously shaking the vine for 30 seconds in order to dislodge spiders into the funnel and plastic collection bag. All spiders were brought to the laboratory where they were identified to family.

### Vine Vigor

Petiole total nitrogen (%) at peak bloom was quantified to assess vine vigor in 2011, 2012 and 2013. Peak bloom was defined as >80% of grape clusters in full bloom. At peak bloom, petioles were collected from 60 randomly selected vines at each site (one petiole per vine). Following Reisenauer [52], each petiole was taken from opposite flower clusters near the base of a shoot. Petioles were brought to the laboratory, washed with deionized water and dried at 55°C for 24 hours. Samples were then sent to the University of California Division of Agriculture and Natural Resources Analytical Laboratory to quantify total nitrogen levels.

### Statistical Analysis

#### Data aggregation and summary

Data from the five yellow sticky-traps at each site were averaged for each sample date and then converted to the number of organisms per day to account for differences in the length of each sample period. Since *Anagrus* populations can exhibit a rapid, density-dependent response to *E*. *elegantula*, data from only the first sample date in each year was used as the measure of early season *Anagrus* densities. For the late season, *Anagrus* density per day was summed across all sample dates in that period. Peak *E*. *elegantula* densities in the early and late season period were determined by selecting the sampling date with the highest density of *E*. *elegantula* per trap per day in each period. Early and late season generalist predator data were summed across all sample dates in each sample period. Data from the five beat samples of the vine canopy at each site were summed for each year of the study, resulting in one measure per site per year.

#### Calculating natural enemy evenness

Community evenness was separately quantified for generalist predators on the sticky-traps in both the early and late season periods as well as for spiders from the beat sampling by calculating Pielou’s *J* (J = H’/ln(S)) using the “vegan” package in the statistics program R (version 3.0.3, *http*:*//www*.*r-project*.*org/*). This was derived by first calculating the Shannon-Weaver index (H’): H’ = - ∑ (P_i_ * ln P_i_) where *P*_*i*_ is the fraction of the entire population made up of species *i*, and then dividing H’ by the logarithm of species richness (S). J falls in a range of 0–1, with higher values representing a more even community.

#### Mixed-effects models

Linear mixed-effects models (“lme4” package) were used to evaluate data on pest and natural enemy densities as well as total petiole nitrogen content. Parasitism data were evaluated with logistic regression. All of the analyses included “Year” and “Site” as crossed random interaction effects. To improve normality, all data on insect densities were log(x+1) transformed and the log odds transformation was used for petiole total nitrogen content (a percentage). Model comparison (χ^2^ tests) was used to evaluate the influence of main effects against a reduced (null) model. When a factor with more than two levels (i.e. cultivar and rootstock) was found to have a significant effect, post-hoc Tukey contrasts (“glht” function in the “multcomp” package) were used in order to make comparisons between multiple factors.

Analysis of early and late season generalist predator densities and evenness, as well as spider family abundance and evenness included the main effects “natural habitat within 0.5 km” and “peak *E*. *elegantula* density” (first generation peak for early season, second generation peak for late season, which includes the spider data). Analysis of total petiole nitrogen content included the main effects “grape cultivar”, “rootstock”, “vine density per acre”, and “natural habitat within 0.5 km”. Early and late season *Anagrus* and *E*. *elegantula* densities and parasitism rates were evaluated against a number of factors, see Table 3 for a summary of all main effects.

**Table 3:** Results from the Analysis of *Anagrus* and *E*. *elegantula* Abundance and Parasitism Rate.

		Early Season			Late Season		
Response	Factor	n	χ^2^	*P*	n	χ^2^	*P*
*Anagrus* density	Cultivar	68	6.14	0.19	56	3.60	0.47
	*E*. *elegantula* density		2.70	0.10		**20.8**	**<0.001**
	Landscape diversity		**5.05**	**0.03**		2.45	0.12
	First generation parasitism		**-**	**-**		0.41	0.53
*E*. *elegantula* density	Cultivar	28	4.64	0.10	18	**14.03**	**<0.001**
	Rootstock		9.15	0.24		**40.43**	**<0.001**
	*E*. *elegantula*—previous year		**7.21**	**<0.01**		-	-
	Total petiole nitrogen content		0.34	0.56		**16.19**	**<0.001**
	Landscape diversity		**7.73**	**<0.01**		3.34	0.07
	Natural enemy evenness (*J*)		0.27	0.60		0.16	0.69
	First generation parasitism		-	-		**3.85**	**0.05**
	Spider evenness (*J*)		-	-		1.32	0.25
Parasitism rate	*Anagrus* x *E*. *elegantula*	55	**5.73**	**0.02**	56	**25.28**	**<0.001**
	Landscape diversity		0.78	0.38		**4.38**	**0.04**
	First generation parasitism		-	-		3.45	0.06

Due to uneven sampling across all sites in all years, the number of complete cases varied in each analysis depending on which main effects were included in the model being evaluated. In general, including more effects reduced the sample size. Sample size is therefore indicated for each analysis. All data used in this analysis are available from the Dryad Digital Repository: http://dx.doi.org/10.5061/dryad.c1m0p [53]

## Results

### Generalist Predator Abundance and Evenness

Early and late season densities and evenness of generalist predators from the yellow sticky-traps and spiders from the beat sampling did not consistently correspond to the proportion of natural habitat within 0.5 km or to *E*. *elegantula* density (Table 4 and Table 5). Analysis of spider densities was based on counts by family, but Table 5 also includes a list of species from each family that are commonly found in the study region, this is based on recent surveys by Hogg and Daane [44, 54–56]. Early season *Chrysoperla* sp. and late season *Orius* sp. densities both positively correlated with *E*. *elegantula* densities. Abundance of spiders in the family Anyphaenidae were increased in vineyards with more natural habitat and Miturgidae density was elevated at sites with high *E*. *elegantula* density.

**Table 4:** Generalist Predator Response to Landscape Diversity and *E*. *elegantula* Density (n = 58).

	Early Season				Late Season			
	Landscape Diversity		*E*. *elegantula* Density		Landscape Diversity		*E*. *elegantula* Density	
Predator	χ^2^	*P*	χ^2^	*P*	χ^2^	*P*	χ^2^	*P*
Cantharidae	0.20	0.66	1.29	0.26	1.05	0.31	0.01	0.95
*Chrysoperla* sp.	0.23	0.63	**6.67**	**0.01**	1.18	0.28	0.71	0.40
*Geocoris* sp.	0.10	0.75	-	-	0.87	0.35	0.29	0.59
*Hemerobius* sp.	0.02	0.89	3.43	0.06	0.59	0.44	1.13	0.29
*H*. *convergens*	2.60	0.11	0.60	044	0.67	0.41	2.28	0.13
*Nabis* sp.	-	-	-	-	-	-	-	-
*Orius* sp.	0.30	0.58	0.02	0.90	2.03	0.16	**10.77**	**<0.01**
Syrphidae	0.29	0.59	1.60	0.20	0.71	0.40	0.05	0.83
Evenness (*J*)	0.69	0.41	0.01	0.93	1.02	0.31	0.01	0.95

**Table 5:** Response of Spiders to Landscape Diversity and *E*. *elegantula* Density (n = 53).

		Landscape Diversity		*E*. *elegantula* Density	
Spider Family	Common Species*	χ^2^	*P*	χ^2^	*P*
Agelenidae	*Hololena nedra*	1.14	0.29	0.97	0.33
Anyphaenidae	*Anyphaena pacifica*	**6.13**	**0.01**	1.44	0.23
	*Hibana incursa*				
Araneidae	*Araneus* spp.	1.13	0.29	1.82	0.18
	*Araniella* spp.				
	*Cyclosa turbinata*				
	*Metepeira* spp.				
	*Nuctenea* sp.				
Corinnidae	*Castianeira* sp.	1.49	0.22	0.13	0.72
	*Meriola californica*				
	*Trachelas pacificus*				
Desidae	*Badumna longinqua*	0.55	0.46	1.10	0.29
Dictynidae	*Dictyna* spp.	0.01	0.98	0.02	0.89
	*Mallos* sp.				
Gnaphosidae	*Micaria* sp.	2.16	0.14	-	-
	*Zelotes* spp.				
Linyphiidae	*Erigone* spp.	0.01	0.97	0.56	0.46
	*Pityohyphantes* sp.				
Lycosidae	*Hogna* sp.	0.11	0.74	0.03	0.87
	*Pirata* sp.				
Miturgidae	*Cheiracanthium inclusum*	0.16	0.69	**4.94**	**0.03**
	*Cheiracanthium mildei*				
Oxyopidae	*Oxyopes salticus*	0.71	0.40	0.19	0.66
	*Oxyopes scalaris*				
	*Oxyopes* sp.				
Salticidae	*Habronattus* sp.	0.05	0.82	0.54	0.46
	*Menemerus bivattatis*				
	*Metaphidippus manni*				
	*Metaphidippus* sp.				
	*Phidippus* spp.				
	*Salticus scenicus*				
	*Sassacus vitis*				
	*Thiodiona hespera*				
Tetragnathidae	*Tetragnatha laboriosa*	-	-	-	-
	*Tetragnatha versicolor*				
Theridiidae	*Euryopis* sp.	0.17	0.68	1.07	0.30
	*Theridion dilutum*				
	*Theridion melanurum*				
	*Theridion* spp.				
Thomisidae	*Coriarachne brunneipes*	2.36	0.12	0.01	0.98
	*Diaea* sp.				
	*Misumena vatia*				
	*Misumenops* spp.				
	*Tmarus* sp.				
	*Xysticus gulosus*				
Unknown		0.09	0.77	0.01	0.95
Evenness (*J*)		3.33	0.07	0.70	0.40
Total		0.05	0.82	1.81	0.18

*Common species are based on previous surveys in this region [44, 54–56].

### *Anagrus* and *E*. *elegantula* Abundance

**Fig 1:**
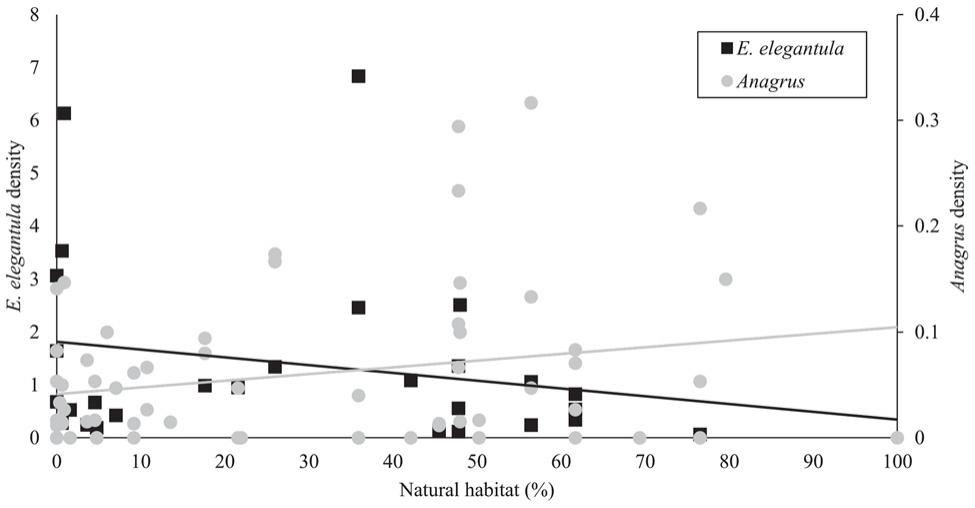
Early in the season, vineyards in more diverse landscapes had higher *Anagrus* densities and lower *E*. *elegantula* densities.

**Fig 2:**
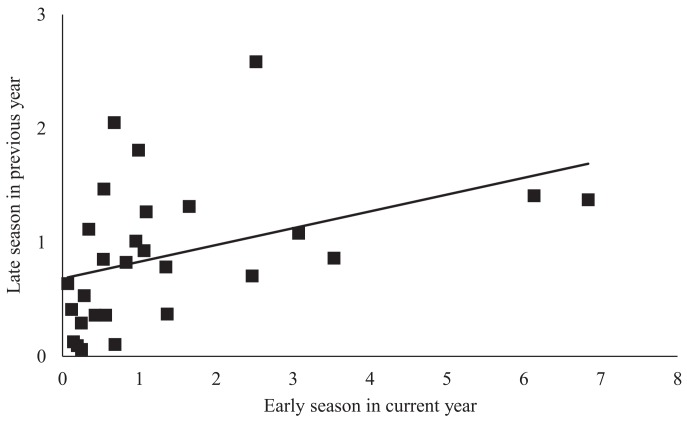
Current year early season density of *E*. *elegantula* is determined by late season density in the previous year.

**Fig 3:**
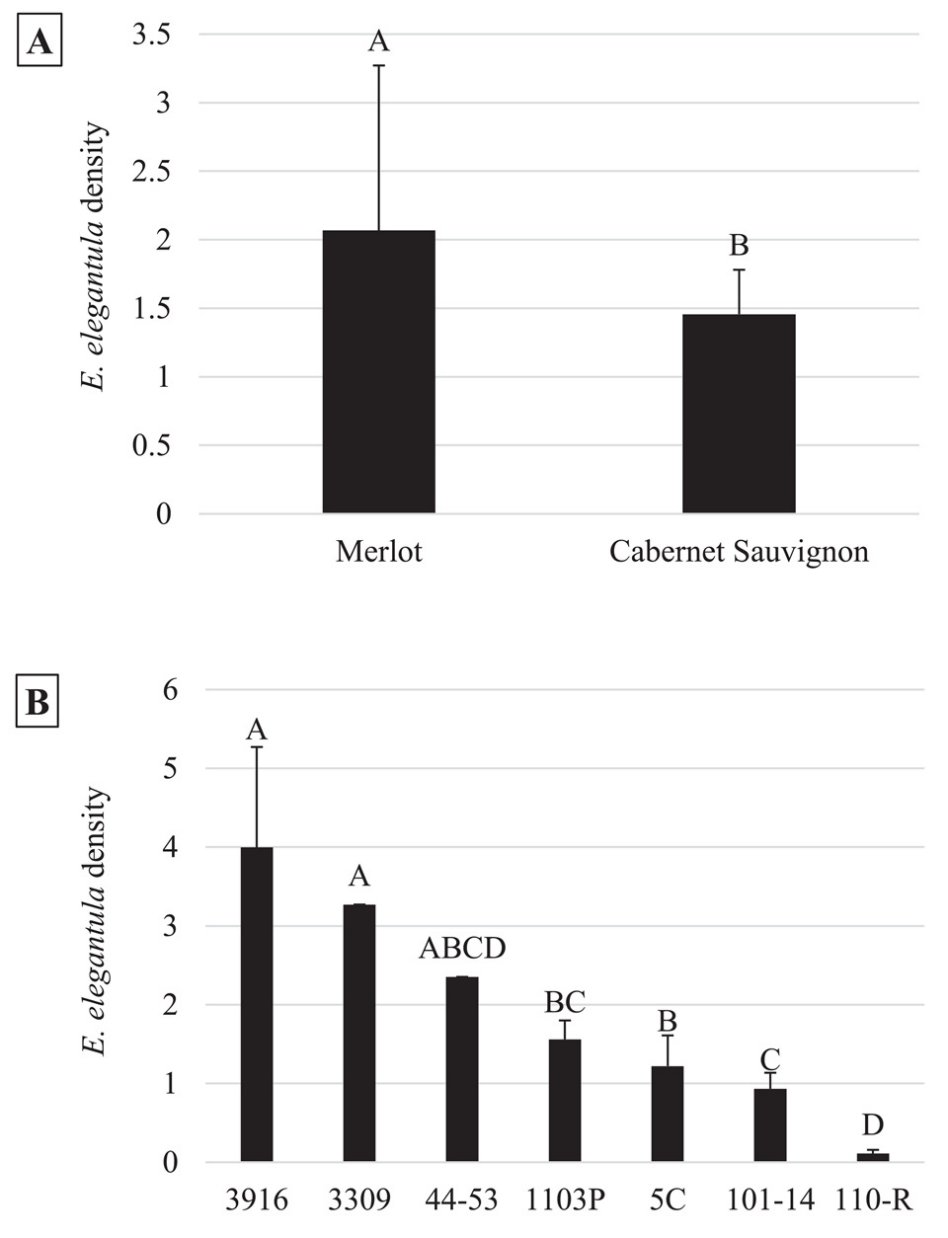
Peak second generation *E*. *elegantula* densities were influenced by grape cultivar (3a) and rootstock (3b).

**Fig 4:**
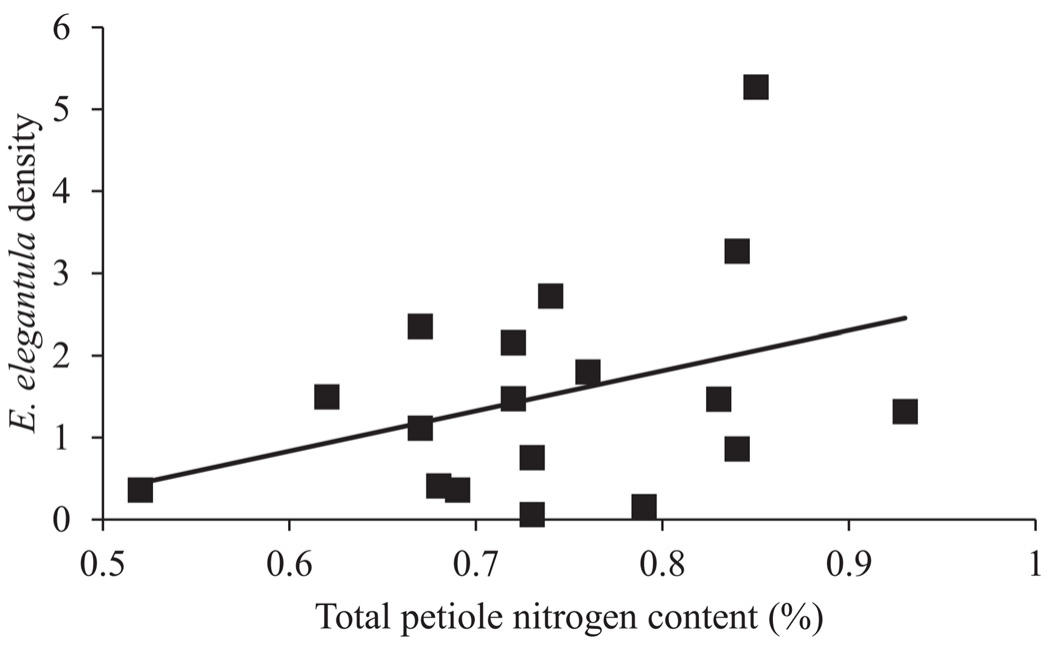
Peak second generation *E*. *elegantula* densities were higher on vines with increased total petiole nitrogen content.

**Fig 5:**
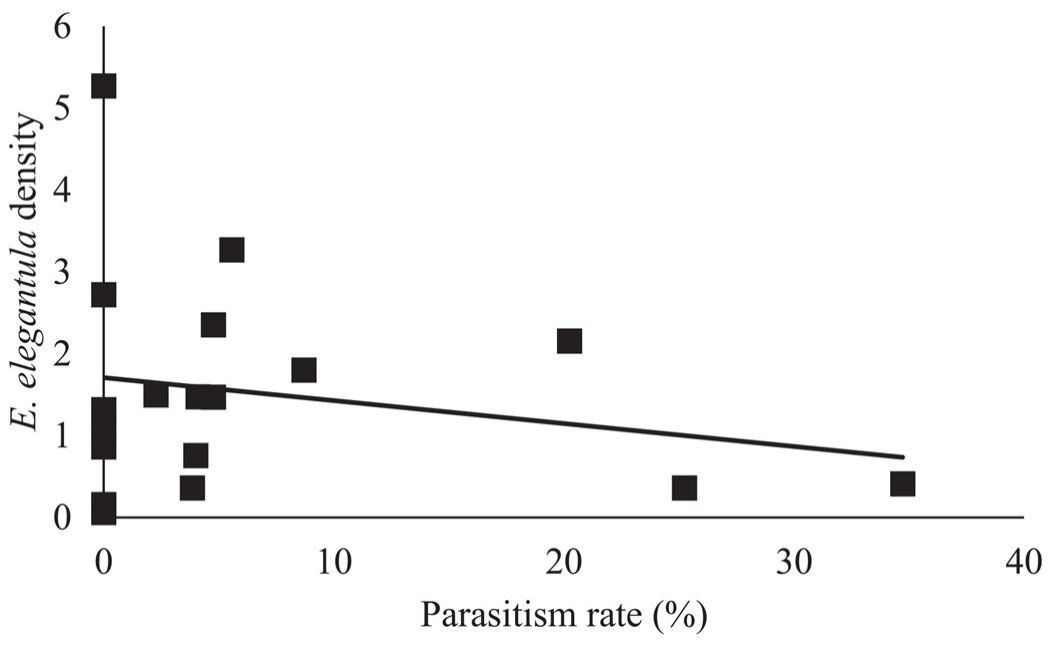
Peak second generation *E*. *elegantula* was lower at sites with high first generation parasitism rates.

In the early season, sites with increased natural habitat had higher *Anagrus* densities (n = 28, χ^2^ = 5.05, *P* = 0.03) and lower *E*. *elegantula* densities (n = 28, χ^2^ = 7.73, *P* < 0.01) (Fig 1). Early season densities of *E*. *elegantula* were also higher at sites with high late season densities in the previous year (n = 28, χ^2^ = 7.21, *P* < 0.01) (Fig 2). Late season abundance of *Anagrus* closely correlated with peak *E*. *elegantula* density during this period (n = 56, χ^2^ = 20.77, *P* < 0.001). Late season densities of *E*. *elegantula* were strongly related to grape cultivar (n = 18, χ^2^ = 14.03, *P* < 0.001) (Fig 3A) and rootstock (n = 18, χ^2^ = 40.43, *P* < 0.001) (Fig 3B) and were higher at sites with increased total petiole nitrogen content (n = 18, χ^2^ = 16.19, *P* < 0.001) (Fig 4) and lower at sites with increased first generation parasitism rate (n = 18, χ^2^ = 3.85, *P* = 0.05) (Fig 5). See Table 3 for a summary of the full analysis, including those factors that did not have a significant effect on *Anagrus* or *E*. *elegantula* densities.

### Parasitism of *E*. *elegantula* Eggs

**Fig 6:**
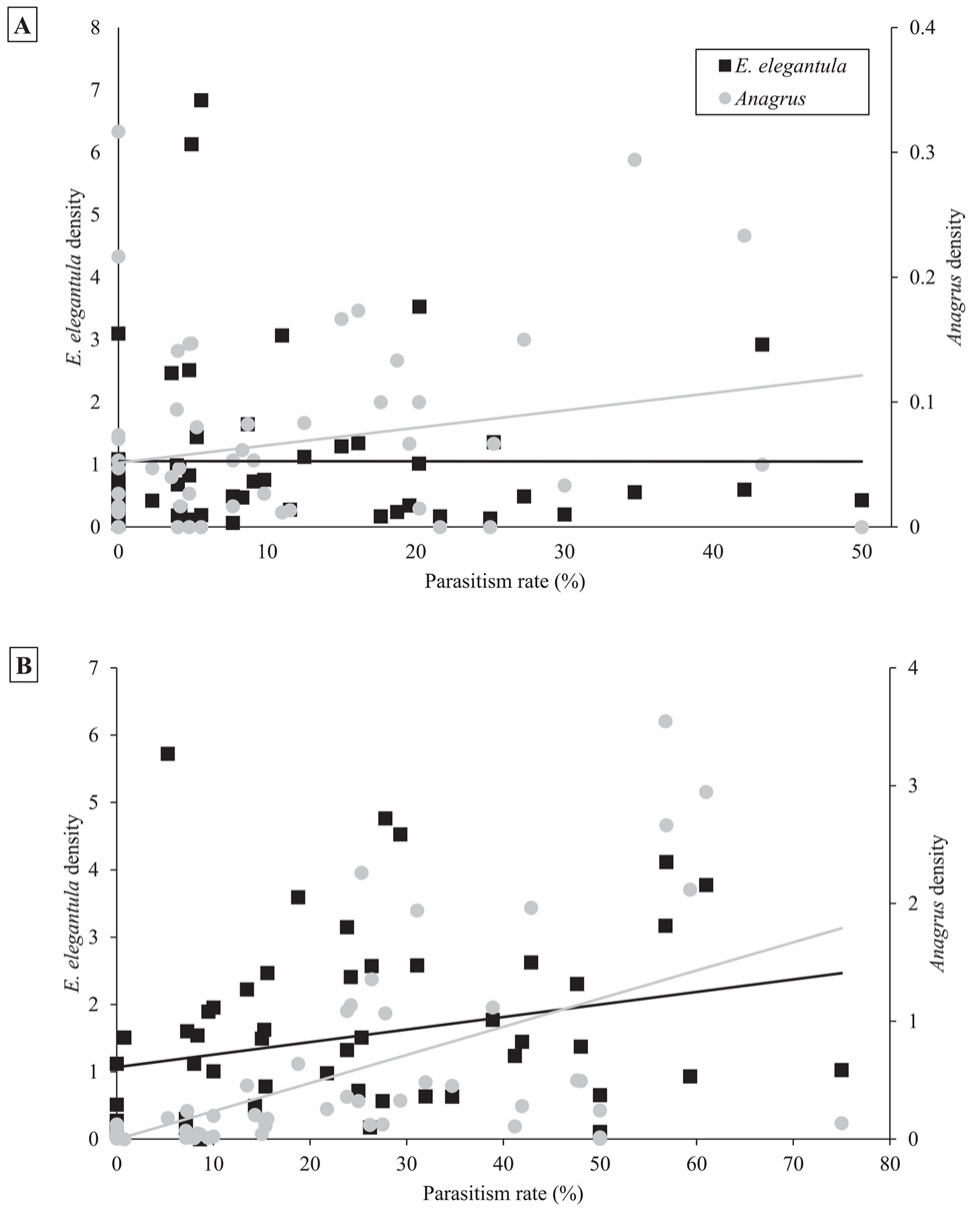
Higher ratios of *Anagrus* to *E*. *elegantula* led to increased parasitism of both first generation (6a) and second generation (6b) *E*. *elegantula* eggs.

Parasitism of both first and second generation *E*. *elegantula* eggs was determined by an interaction effect between *Anagrus* and *E*. *elegantula* densities (first generation n = 55, χ^2^ = 5.73, *P* = 0.02; second generation n = 56, χ^2^ = 25.28, *P* < 0.001) (Fig 6). Second generation parasitism was also negatively influenced by natural habitat (n = 56, χ^2^ = 4.38, *P* = 0.04). Landscape diversity did not impact parasitism rates in the first generation and first generation parasitism did not impact parasitism rates in the second generation (Table 3).

### Vine Vigor

**Fig 7:**
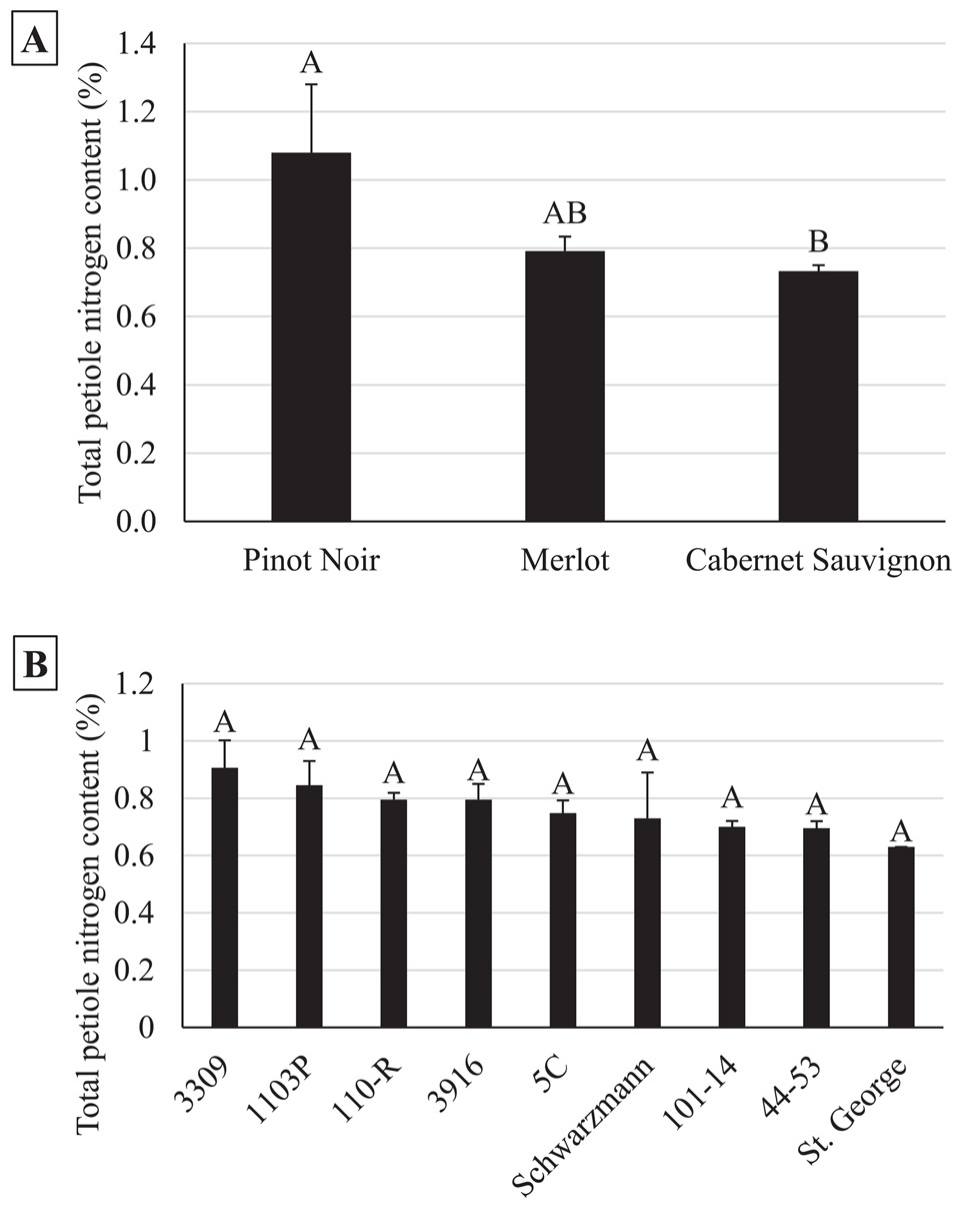
Total petiole nitrogen content varied by cultivar (7a) but not rootstock (7b).

Total petiole nitrogen content varied with grape cultivar (n = 40, χ^2^ = 7.42, *P* = 0.02) (Fig 7A), but did not appear to be influenced by rootstock (n = 40, χ^2^ = 9.85, *P* = 0.28) (Fig 7B), vine density (n = 40, χ^2^ = 1.25, *P* = 0.26), or the amount of natural habitat surrounding the vineyard (n = 40, χ^2^ = 0.02, *P* = 0.89).

## Discussion

### Spiders and Other Generalist Predators

For the most part, generalist predator response to changes in landscape diversity follows Thomson et al. [46] and D’Alberto and Hoffman [45] who both found weak or inconsistent trends. Spiders in the family Anyphaenidae were an exception to this and matches findings from Hogg and Daane [44], who proposed that native spiders found in vineyards might be more closely associated with natural habitats. Alternately, it may be that spider response varies by functional guild, as demonstrated by Isaia et al. [43]. Data from this current study do not fully support either hypothesis though, as there was no uniform response across native spiders or any specific functional guild. The lack of a strong relationship between landscape diversity and generalist predator densities may be indicative of their less than obligate relationship with natural habitats, which has been cited as a key determinant of insect response to changes in the area of natural habitats surrounding an agroecosystem [5].

Alternately, *Chrysoperla* sp., *Orius* sp., and spiders in the family Miturgidae all appeared to demonstrate a functional response to increased *E*. *elegantula* densities at some point over the season. This is not uncommon for generalist predators [57], but whether or not this had a significant impact on *E*. *elegantula* densities is unclear, as no evaluation of predation pressure was conducted.

### *Anagrus* and *E*. *elegantula* Density–Early Season

Increased densities of *Anagrus* is likely due to the proximity of parasitoid overwintering habitat found in natural habitats, as observed in previous studies [38, 41]. The effect of previous year population on leafhopper abundance follows de Valpine et al. [58] who observed a similar year-to-year trend in vineyard leafhopper densities. Landscape diversity and pest densities in the previous year could act in conjunction to determine populations in vineyards the following year. Low abundance of *E*. *elegantula* at the end of the year could lead to a smaller overwintering population, which in a high diversity landscape is then further subject to increased predation during the winter due to increased natural enemy populations associated with the natural habitats surrounding the more diverse vineyards. For example, Eilers and Klein [59] found that overwintering navel orangeworm (*Amyelois transitella* Walker, Lepidoptera: Pyralidae) was more likely to be attacked by natural enemies in orchards with higher levels of surrounding natural habitat.

### Parasitism of *E*. *elegantula*

First and second generation parasitism rate of *E*. *elegantula* eggs was most strongly and consistently determined by the ratio of *Anagrus* to *E*. *elegantula*, which itself was determined by landscape diversity. Lower second generation parasitism rates at the more diverse sites may just be the consequence of generally lower *E*. *elegantula* densities at these sites to begin with. While natural habitats may serve as a consistent source of *Anagrus* that continuously migrate into the vineyard throughout the entire season, it may be that this effect was diluted by a density dependent response of *Anagrus* to increased early season *E*. *elegantula* densities at the less diverse sites. While increased parasitoid abundance and parasitism rates in more diverse landscapes has been demonstrated in a number of studies [1, 7, 60], in this case the link between landscape diversity and parasitism rate is more indirect.

### *Anagrus* and *E*. *elegantula* Density–Late Season

Increased late season *Anagrus* densities is likely due to a density dependent response to increased availability of *E*. *elegantula* hosts (data not shown). Peak second generation density is arguably the most important measure of *E*. *elegantula* pressure in this agroecosystem. This is because growers typically determine the need to apply chemical control measures for this pest based on population assessments at this time of the season [31]. The influence of cultivar and rootstock on *E*. *elegantula* densities is likely due to a combination of differences in the structural and chemical characteristics of the vine. For example, McKenzie and Beirne [61] found that structural differences between grape varieties influenced *E*. *ziczac* populations, which preferred to feed and reproduce on vines with more glabrous leaves. Leafhopper preference for more glabrous crop varieties has been demonstrated in other cropping systems as well [62–64]. Here, the difference in *E*. *elegantula* densities observed between Cabernet Sauvignon and Merlot may be due to a similar effect, although both are considered to have sparse leaf hairs. Alternately, Settle et al. [29] found that late season *E*. *variabilis* densities were higher on grape vines grafted to more vigorous rootstock. This was attributed to leafhopper preference for vines with increases nitrogen levels, which has been demonstrated for multiple *Erythroneura* species on grape vines [65, 66]. Leafhopper preference for specific rootstocks has been observed in other perennial systems as well [67, 68]. Surprisingly, total petiole nitrogen content was found to be more contingent upon grape cultivar than rootstock (Fig 7).

Nitrogen is one of many nutrients important for insect development, and although *E*. *elegantula* did respond to nitrogen, it may not adequately represent the specific changes in plant chemistry that led to increased *E*. *elegantula* densities on certain rootstocks. For instance, previous studies have shown that rootstocks can influence a number of different nutrients in grape tissue [69–71] and that leafhoppers respond not only to changes in nitrogen levels but rather a range of plant metabolites [28, 72–75]. Regarding parasitism, the rapid development rate of *Anagrus* allows it to complete up to three generations over the course of the *E*. *elegantula* egg maturation period [76]. As such, densities can exponentially increase in a short amount of time and a high rate of parasitism in the early season is commonly linked to increased late season parasitism, as was observed here [31, 38].

## Conclusions

One of the drivers of lower second generation *E*. *elegantula* densities was increased first generation parasitism. First generation parasitism was found to be related to the ratio of *Anagrus* to *E*. *elegantula* densities in the early season, which itself was influenced by the proportion of natural habitat surrounding a vineyard. As such, there appears to be a series of linkages between landscape diversity, early season abundance of *Anagrus* and parasitism of *E*. *elegantula*, and ultimately second generation *E*. *elegantula* densities. Additionally, *E*. *elegantula* densities were also influenced by cultivar, rootstock and vine vigor. This is likely due to ways in which the physical and chemical properties of specific grape cultivars and rootstocks influence host-plant quality and *E*. *elegantula* preference and performance. Additional work is necessary to quantify differences in vine nutrient composition associated with various cultivar-rootstock combinations as well as *E*. *elegantula* response to such changes. While increased landscape diversity can lead to increased parasitism of *E*. *elegantula* by *Anagrus*, pest densities are also influenced by crop characteristics like cultivar, rootstock and vigor.
